# Supplementary material for: Socio-economic disparities and predictors of fertility among adolescents aged 15 to 19 in Zambia: Evidence from the Zambia Demographic and Health Survey (2018)
Source: PLOS Glob Public Health. 2024 Mar 27;4(3):e0002739. doi: 10.1371/journal.pgph.0002739 (PMC10971614; doi:10.1371/journal.pgph.0002739)
Supplement: Attachment — Submitted filename: Response to Reviewers.docx [file pgph.0002739.s001.docx]

Department of Epidemiology and Biostatistics

School of Public Health,

The University of Zambia,

Lusaka, Zambia.

29^th^ January, 2024.

PLOS Global Public Health

Dear Editorial Team,

**Subject: Response to Reviewer Comments on Manuscript ID (PGPH-D-23-02336)**

I hope this correspondence finds you well. We would like to express our gratitude for the insightful comments and feedback provided by the reviewers on our manuscript titled "Socio-economic disparities and predictors of fertility among adolescents aged 15 to 19 in Zambia: Evidence from the Zambia demographic and health survey (2018)" (Manuscript ID: PGPH-D-23-02336). We highly appreciate the time and effort invested in reviewing our work.

After careful consideration of the comments, we have diligently addressed each concern raised by the reviewers. A summary of the major revisions and our responses is outlined below:

1. Specify the independent and dependent variables in the abstract.

Response: The comment has been worked on. The variables have been included in the abstract.

2. Mention few more key words.

Response: two more key words have been added

3. The rationale of this study requires more development. Please review relevant literature, leading to your paper aim.

Response: Introduction has been refined and more literature has been added.

4. How did you select the independent variables? Based on review of the literature? If yes, please state it with citations.

Response: the citations have been included and the statement has been refined.

5. Ethics section can be shorter as you applied the secondary sources of data, which do not require ethics approval.

Response: the statement has been refined.

6. The first paragraph of the discussion section requires revision. Please state and summarize your findings here.

Response: The paragraph has been revised.

7. Delete justification as it is in a wrong place; it should be in the introduction. Rather state the limitations and strengths of your study here.

Response: subtitle has been changed to conclusion and policy implications.

8. Conclusions and policy implications?

Response: The subtitle has been changed to conclusions and implications

**Reviewer 1**

1. Abstract: Missing aim or hypothesis to be tested and details in design.

Response: the aim of the study has been added

2. More detailed statistics are needed after the statement, “Zambia, has grappled with elevated fertility rates…”

Response: statement has been included

3. Serves more as a review of ideas surrounding childbirth and potential individual and community level factors than as a means to allow for deductive reasoning that would allow for a hypothesis to be developed that is necessary for conducting the analysis that you have indicated performing. Ignoring numerous studies have already shown the interaction of childbirth, individual and community level factors. Missing a clear definition of the main variable of interest “fertility,” and how the authors utilize this term in the study.

Response: the statement has been refined and the variable of interest has been stated clearly.

**Methods**

4. Manuscript lacks consistency in the use of key terms, specifically for the population of interest e.g. adolescent’s aged 15–19 versus women.

Response: the statement has been refined.

5. A brief description is needed for the Bongaarts proximate determinants of fertility.

Response; in response, a clarifying statement has been incorporated, and an alternative approach has been introduced for a more comprehensive understanding.

6. Missing sample size details (3,112 only is mentioned in the abstract). The Zambia Demographic and Health Survey seems to have more than 3,112.

Response: that has been clarified.

7. Missing details necessary to explain selection process of data included and excluded or how you obtained information.

Response: Statement has been included in the methods section to include.

8. Missing information if the sample weighting option was used/not used for such national representative data? And why?

Response: In response, the statement has been added patterning to the sampling weight.

9. How were you able to control the existing bias in responses?

Response: to mitigate the recall bias, performed a thorough data cleaning to identify and address inconsistencies or outliers. It is essential to recognize these factors for a nuanced interpretation of the study's findings.

10. Missing explanation of inclusion and exclusion of responses e.g. missing values

Response: The inclusion criteria has been mentioned in the methods section second paragraph, and details regarding the exclusion of responses and management of missing values have been articulated. All files meeting the inclusion criteria were considered in the analysis.

11. Dependent and independent variables: The description and use of the main dependent variable depicts a faulty message, and it may not reflect the actual meaning of the variable, “ever given birth/children ever born”. The variable appears to be more of "Yes, No" question instead of multilevel responses that are used in the manuscript, “No Child, 1 Child, and 2+ Children”, which indicates to the number of children.

Response: the variable is “total number of children ever born”, which constitutes the number of children born to females at the time of survey. The statement has been made clearer than it was.

12. How did you handle measures to account for biased responses?

Response: to mitigate the recall bias, performed a thorough data cleaning to identify and address inconsistencies or outliers. It is essential to recognize these factors for a nuanced interpretation of the study's findings.

13. Missing details on included measures and how they were calculated e.g. wealth index.

Response: the statement has been included.

14. Missing how you established parameters for Chi-square

Response: statement has been included

15. Reporting on odds ratio, but no indication for calculating an odds ratio or how you went about adjust the odds ratio to indicate why you needed to adjust that odds ratio.

Response: the regression model used is Mixed Effect Ordinal Logistic regression which produces estimates as odds ratios. Moreover, the adjusted odds ratio implies the odds ratio in a model with explanatory variables.

Results:

16. Cannot critically analyze report based on faulty/unclear utilization of variables, specifically the variable of interest “fertility”.

Response: the statement has been refined to give more light to the variable of interest. In support to this, a study was similarly done in Kenya and is cited below in which they used the same outcome variable:

Monari N, Orwa J, Agwanda A (2022) Adolescent fertility and its determinants in Kenya: Evidence from Kenya demographic and health survey 2014. PLoS ONE 17(1): e0262016. https:// doi.org/10.1371/journal.pone.0262016

17. Tables are clear and easy to follow.

Response: noted

18. Model 1 was labeled as “AOR”, however, it is unclear whether this model was adjusted for other variables.

Response: It was a typo and has been corrected to “OR”, because it has no explanatory variables.

19. Have you considered using the age specific fertility rate (ASFR) or average/mean number of children ever born (MCEB) instead of just number of children?

Response: he statement has been enhanced to provide additional clarity regarding the outcome variable. Opting for the standard outcome variable "total number of children ever born" is deemed more practical for analysis compared to utilizing the Age-Specific Fertility Rate (ASFR). This decision is supported by a similar methodological approach employed in a referenced study conducted in Kenya by Monari et al. (2022), where the chosen analytical method is justified. (Monari N, Orwa J, Agwanda A (2022) Adolescent fertility and its determinants in Kenya: Evidence from Kenya Demographic and Health Survey 2014. PLoS ONE 17(1): e0262016. https://doi.org/10.1371/journal.pone.0262016)

**Discussion:**

20. Cannot critically discuss due to faulty/unclear utilization of variables and subsequently analysis and results being offered.

Response: the statement has been refined and more details have been added to it.

21. Missing the inductive reasoning that would provide a means to draw a conclusion and offer insight into how findings fit into/contribute to the larger base of knowledge that we already have.

Response: The section has been refined, to ensure that it is clearer.

22. More results need to be emphasized, discussed and compared e.g. children ever born vs wealth index.

Response: the relationship of total number of children ever born vs wealth index is there in the descriptive table and the interpretation was done.

23. Biased responses were not indicated in the limitation.

Response: statement has been added in the limitations

24. Missing detailed information of current reproductive health policies, and recommended policies to be implemented.

Response: current policies have been included, recommended policies were already included and are in the policy implications but have been synchronized with the existing current policies.

**Reviewer #2:**

1. The article provides substantial information regarding the topic. There are a couple of sentences which need to be referenced. For instance, 'Studies have shown that adolescent mothers face high risks of eclampsia…

Response: statement has been refined.

We believe that the revisions made have significantly strengthened the quality and robustness of our manuscript. We are confident that the changes align with the high standards set by PLOS Global Public Health and enhance the overall contribution of our work to the scientific community.

We kindly request a reevaluation of our revised manuscript and hope that the changes will be deemed satisfactory. We are committed to ensuring the highest level of accuracy, clarity, and scientific rigor in our submission.

Thank you for your time and consideration.

Sincerely,

Samson Shumba,

The University of Zambia,

[Samsonshumba1@gmail.com](mailto:Samsonshumba1@gmail.com).

Socio-economic disparities and predictors of fertility among adolescents aged 15 to 19 in Zambia: Evidence from the Zambia demographic and health survey (2018)

PGPH-D-23-02336
